# Neuroprotective Role of Akt in Hypoxia Adaptation in Andeans

**DOI:** 10.3389/fnins.2020.607711

**Published:** 2021-01-15

**Authors:** Helen Zhao, Jonathan Lin, Gary Sieck, Gabriel G. Haddad

**Affiliations:** ^1^Department of Pediatrics (Respiratory Medicine), University of California, San Diego, La Jolla, CA, United States; ^2^Department of Pathology, University of California, San Diego, La Jolla, CA, United States; ^3^Department of Pathology, Stanford University, Stanford, CA, United States; ^4^VA Palo Alto Healthcare System, Palo Alto, CA, United States; ^5^Department of Physiology and Biomedical Engineering, Mayo Clinic, Rochester, MN, United States; ^6^Department of Neurosciences, University of California, San Diego, La Jolla, CA, United States; ^7^The Rady Children’s Hospital, San Diego, CA, United States

**Keywords:** chronic mountain sickness, iPSCs, neurons, Akt, Parkin, hypoxia, cell death

## Abstract

Chronic mountain sickness (CMS) is a disease that potentially threatens a large segment of high-altitude populations during extended living at altitudes above 2,500 m. Patients with CMS suffer from severe hypoxemia, excessive erythrocytosis and neurologic deficits. The cellular mechanisms underlying CMS neuropathology remain unknown. We previously showed that iPSC-derived CMS neurons have altered mitochondrial dynamics and increased susceptibility to hypoxia-induced cell death. Genome analysis from the same population identified many ER stress-related genes that play an important role in hypoxia adaptation or lack thereof. In the current study, we showed that iPSC-derived CMS neurons have increased expression of ER stress markers Grp78 and XBP1s under normoxia and hyperphosphorylation of PERK under hypoxia, alleviating ER stress does not rescue the hypoxia-induced CMS neuronal cell death. Akt is a cytosolic regulator of ER stress with PERK as a direct target of Akt. CMS neurons exhibited lack of Akt activation and lack of increased Parkin expression as compared to non-CMS neurons under hypoxia. By enhancing Akt activation and Parkin overexpression, hypoxia-induced CMS neuronal cell death was reduced. Taken together, we propose that increased Akt activation protects non-CMS from hypoxia-induced cell death. In contrast, impaired adaptive mechanisms including failure to activate Akt and increase Parkin expression render CMS neurons more susceptible to hypoxia-induced cell death.

## Introduction

Chronic Mountain Sickness (CMS) is a maladaptation to hypoxia that is characterized by severe hypoxemia, excessive erythrocytosis and many neurologic manifestations, including migraine, headache, mental fatigue, confusion, and memory loss. CMS disease threatens a large segment of the high-altitude population (more than 100 million highlanders) during extended living at altitudes above 2,500 m. Since hypoxia is a common feature of many diseases at sea level including cardiovascular diseases, neurodegenerative diseases, stroke, diabetes and cancer, we believe that investigating the underlying mechanisms of hypoxia maladaptation or adaptation in CMS and non-CMS subjects may lead us to a better understanding of many hypoxia-related diseases as well. However, due to sample availability and ethical issues, our understanding of CMS neuropathology in humans remains largely unexplored at cellular and molecular levels. By using CMS disease-specific iPSC-derived neurons, we have previously reported mitochondrial dysfunction in CMS neurons under normoxia and an increased vulnerability to hypoxia-induced cell death after 48 h (Zhao et al., 2018). Here, we are interested in investigating the underlying mechanisms that contribute to hypoxia-induced cell death in CMS neurons.

Recent clinical studies have shown that CMS patients have blunted cerebral blood flow and exaggerated systemic oxidative-inflammatory-nitrosative stress with impaired cognition (Bao et al., 2017; Bailey et al., 2019) and CMS is believed to have clinical manifestations of aging at high altitude (Sime et al., 1975). In the brain, reduced cerebral blood flow and hypoxia disturb normal physiological functions and lead to metabolic stress. In conditions of stress including hypoxia, organisms activate endogenous adaptive cytoprotection mechanisms including the *endoplasmic reticulum* (ER) unfolded protein response (ER stress response), ER associated degradation (ERAD), autophagy, mitophagy, and/or mitochondrial biogenesis to restore cellular homeostasis (Senft and Ronai, 2015; Wu and Chen, 2015; Xu et al., 2015; Maekawa and Inagi, 2017; Delbrel et al., 2018; Yamashita and Kanki, 2018). Indeed, under pathological conditions, when an element of the endogenous adaptive cytoprotection mechanisms is impaired, the overall cellular homeostasis can be disturbed (Senft and Ronai, 2015).

Considering that the ER and mitochondria form a direct physical contact *via* mitochondria associated membrane (MAM) and functionally interact with each other (Bouman et al., 2011; Senft and Ronai, 2015), we hypothesize that mitochondrial dysfunction might therefore affect ER function as well as other endogenous adaptive mechanisms such as mitophagy. Akt is a pro-survival signaling protein and the PI3K/Akt/mTORC pathway cross links with both ER stress and autophagy (Qin et al., 2010; Kabir et al., 2018). In CMS patients, the Akt gene is shown to associate with chronic mountain sickness (Buroker et al., 2017) and plays an important role in regulating apoptosis in CMS patients with excessive erythrocytosis (Li et al., 2017; Zhao et al., 2017a). Therefore, in the current study, we explored the relationship between mitochondria and ER stress in iPSCs-derived CMS neurons and non-CMS neurons and investigated the potential rescue role of Akt from progression to hypoxia-induced cell death in CMS neurons.

## Materials and Methods

### Ethics Statement

All non-CMS (*n* = 3) and CMS (*n* = 3) subjects in the current study were adult males residing in the Andean mountain range, in Cerro de Pasco, Peru, at an elevation of more than 4,300 m. Each subject signed an informed written consent under protocols approved by the University of California San Diego and the Universidad Peruana Cayetano Heredia, and all experiments were performed in accordance with the relevant guidelines and regulations.

### iPSCs-Derived Neuronal Culture

Detailed experimental procedures were previously described (Zhao et al., 2015). In brief, fibroblast cells obtained from individual’s skin biopsies were reprogrammed into iPSCs using retrovirus containing the Yamanaka factors (*OCT4*, *SOX2*, *KLF4*, and *c-MYC* human cDNAs) (Takahashi et al., 2007). iPSCs-derived neural progenitor cells (NPCs) were generated following an embryoid bodies (EBs) spin protocol (Kim et al., 2011b) with an AggreWell plate (Stem Cell Technologies, Canada) following the manufacturer’s instructions. Rosette-bearing EBs were manually removed and dissociated into individual cells, which were then plated into a poly-L-ornithine/laminin coated plate to generate a monolayer of NPC culture. NPCs were expanded in DMEM/F12 medium containing 1 × glutamax, 0.5 × N2, 0.5 × B27, 1% penicillin/streptomycin, and 20 ng/ml FGF2. Neural terminal differentiation was initiated by withdrawing FGF2 from DMEM/F12 medium when cells reach to 70% confluence. Most of the iPSCs-derived neurons using the current protocol were glutamatergic (>90%) neurons (Zhao et al., 2015).

### Hypoxia Treatment

All cultures were maintained in an incubator supplied with 21% O_2_ and 5% CO_2_ at 37°C. Three-week-old neurons were exposed to hypoxia (1% O_2_) for 6, 24, or 48 h.

### Chemical Treatment

iPSCs-derived neurons were treated with an ER stress inducer either 1 μM thapsigargin (TG, Sigma-Aldrich, #T9033) or 5 μg/ml tunicamycin (TM, Sigma-Aldrich, #T7765) for 24 h as previously described (Oslowski and Urano, 2011). 100 nM Insulin (Roche, #11376497001) was added 30 min before the hypoxia treatment and 1 mM of 4-Phenylbutyric acid (4-PBA, Sigma-Aldrich, #P21005) was added at the beginning of hypoxia exposure (Liu et al., 2016). 5 μM MK2206 (Cayman #1032350-13-2) was added together with hypoxia treatment (Lin et al., 2018).

### Western Blot

Neurons were homogenized with a glass-teflon homogenizer (Thomas Scientific) in RIPA buffer (Cell signaling #9806S), plus protease and phosphatase inhibitors including 1 mM phenylmethylsulfonyl fluoride (Sigma-Aldrich # P7626) and 1 × protease inhibitor cocktail (Thermo Scientific #1861284). Homogenates were then centrifuged for 10 min at 10,000 × *g* and 4°C. The supernatants were collected and protein concentration was determined using a Bio-Rad protein assay kit (Bio-Rad). Twenty micrograms of protein were separated on NuPAGE^TM^ 4–12% Bis-Tris Mini Protein Gel (Invitrogen #NP0321) and then transferred to polyvinylidene difluoride membranes (Millipore #IPVH00010). The membranes were probed with primary antibody in PBST with 5% BSA overnight at 4°C and followed by an appropriate horseradish peroxidase-conjugated secondary antibody (Thermofisher Scientific #A16160 and #65-6120). Immunoreactive bands were visualized using Bio-Rad ChemiDoc XRS with enhanced chemiluminescence (Perkin–Elmer). Equal loading was assessed using GAPDH and data were analyzed using ImageLab software (version 3.0, Bio-Rad). TM- and TG- treated cells act as positive control for ER stress. CCCP-treated PC3 cell lysates (Cell signaling, #21107s) was used as positive control for the western blot of PINK1 and phospho-Ubiquitin (Ser65).

The primary antibodies PERK (1:100, #sc-377400), Grp78 (1:100, #sc-376768), Akt (1:200, #sc-5298), Parkin (1:200, #sc-32282), and Mfn2 (1:200, #sc-100560) were purchased from Santa Cruz Biotechnology. Phospho-PERK (pPERK, 1:250, #649401) and XBP1s (1:500, #619502) were purchased from Biolegend. Phospho-Akt (pAkt, 1:1000, #4060), cleaved PARP (1:1000, #9541), CHOP (1:1000, #2895), PINK1 (1:1000, #6946) and GAPDH (1:1000, #2118) were purchased from Cell Signaling. Phospho-Ubiquitin (Ser65) (pS65-Ub, 1:1000, #ABS1513-l) was obtained from Millipore.

### Lentivirus Transduction

The pLV [Exp]-EGFP: T2A: Puro-EF1A > hPRKN (NM_004562.3) construct was commercially generated by Vector Builder. The transduction was performed on NPCs and successfully transduced NPCs were selected with 1 μg/ml of puromycin (Clontech #631306). Parkin overexpression was confirmed by western blot.

### Statistics

Data were collected from three individuals in each group and from three experiments were performed in each individual. Results were expressed as group mean ± SD. Differences in means were considered statistically significant when *p* < 0.05 by independent *t* tests or one-way ANOVA followed by Holm–Bonferroni *post hoc* analysis as appropriate.

## Results

### Increased ER Stress in CMS Neurons

**FIGURE 1:**
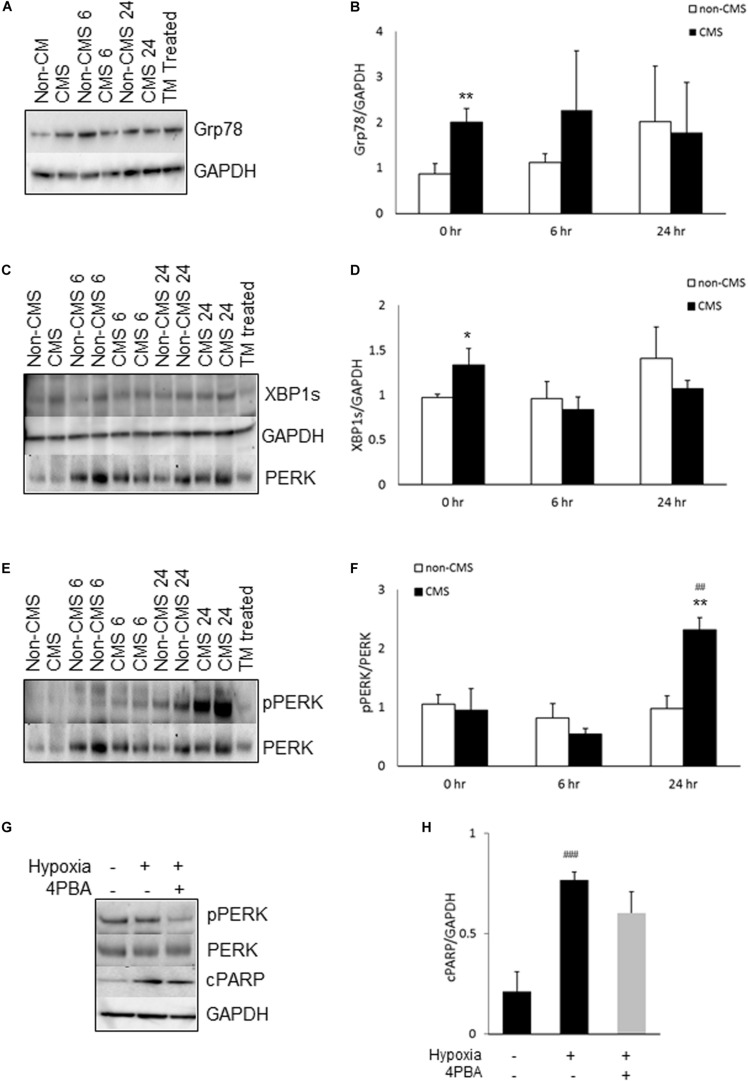
ER stress in CMS neurons. **(A)** A representative blot of Grp78 in iPSC-derived non-CMS neurons (*n* = 3) and CMS neurons (*n* = 3) following hypoxia (1% O_2_) treatment for 0, 6, and 24 h. Tunicamycin (TM)-treated CMS neurons as a positive control for ER stress. **(B)** Densitometry analysis of Grp78. **(C)** A representative blot of XBP1s in iPSC-derived non-CMS neurons (*n* = 3) and CMS neurons (*n* = 3) following hypoxia (1% O_2_) treatment for 0, 6, and 24 h. **(D)** Densitometry analysis of XBP1s**. (E)** A representative blot of PERK in iPSC-derived non-CMS neurons (*n* = 3) and CMS neurons (*n* = 3) following hypoxia (1% O_2_) treatment for 0, 6, and 24 h. **(F)** Densitometry analysis of PERK. **(G)**. A representative blot of PERK and cPARP in iPSC-derived CMS neurons following hypoxia (1% O_2_) treatment for 0 and 48 h with or without 1 mM 4-PBA. **(H)** Densitometry analysis of cPARP. * indicates *p* < 0.05 and ** indicates *p* < 0.01 as compared to non-CMS neurons at a given time point. ^##^ indicates *p* < 0.01 and ^###^*p* < 0.001 as compared to themselves under normoxia.

We have previously reported that CMS neurons had altered mitochondrial dynamics and function (Zhao et al., 2018). Due to the fact that ER and mitochondria form physical contact through MAM and interact with each other functionally (Bouman et al., 2011; Senft and Ronai, 2015), we examined whether the altered mitochondrial function activates the ER stress response. We first measured the expression of Grp78, a well-known marker for ER stress. Grp78 was indeed significantly increased in CMS neurons under normoxia and no difference was detected between non-CMS and CMS neurons under hypoxia (Figures 1A,B, ^∗∗^*p* < 0.01). Under stress conditions, accumulation of unfolded protein dissociates Grp 78 from ER stress sensor proteins, which activates ER stress signaling pathways including IRE1, activating transcription factor-6 (ATF6) and PERK. Both IRE1 and PERK pathways are widely explored and play a critical role in cell death (Szegezdi et al., 2006; Iurlaro et al., 2017). We therefore focused on these two pathways and compared the expression of XBP1s, marker of inositol-requiring protein-1 (IRE1) pathway, and pPERK/PERK, markers of protein kinase RNA (PKR)-like ER kinase (PERK) pathway, in both CMS and non-CMS groups. A significantly higher XBP1s expression was observed in CMS neurons (Figures 1C,D, ^∗^*p* < 0.05), indicating an increased IRE1-mediated ER stress response in CMS neurons under normoxia. The level of PERK phosphorylation (pPERK/PERK) is not different between non-CMS and CMS neurons under normoxia. However, a significantly higher extent of phosphorylation PERK was observed in CMS neurons after hypoxia treatment for 24 h (Figures 1E,F, ##*p* < 0.01) and the difference between non-CMS and CMS was significant (Figures1 E,F, ^∗∗^*p* < 0.01), suggesting a hyperactivation of the PERK pathway of the ER stress response in CMS neurons under hypoxia.

To examine whether hypoxia-induced hyperactivation of the PERK pathway eventually results in an increased cell death of CMS neurons, we treated CMS neurons with the ER stress alleviator 4-Phenylbutyric acid (4-PBA) and exposed them to hypoxia for 48 h, and then measured the expression of cleaved PARP (cPARP), an apoptosis marker (Koh et al., 2005). As shown in Figures 1G,H, 4-PBA treatment indeed inhibited phosphorylation of PERK (∼ 61% decrease, *p* < 0.05) and 4-PBA treatment has a tendency to rescue hypoxia-induced cell death, but the *p* value did not reach statistical significance (Figure 1H, *p* = 0.08).

### Hypoxia Induces Akt Activation in Non-CMS Neurons

**FIGURE 2:**
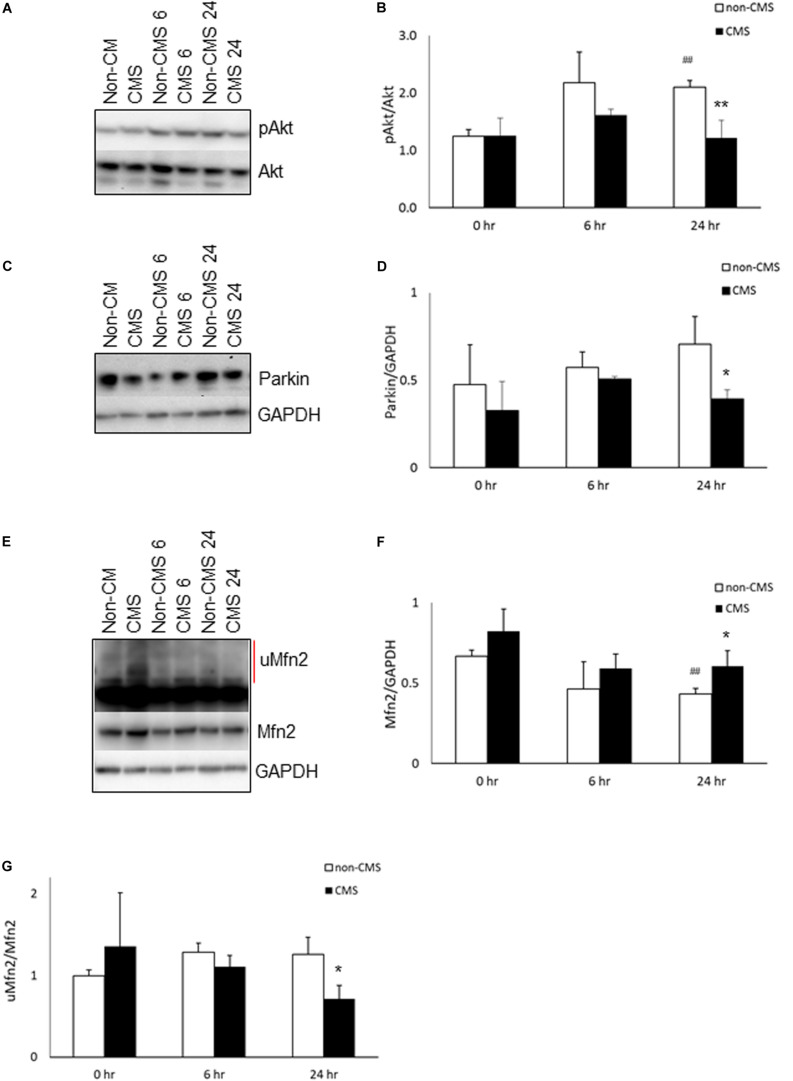
Akt activation in CMS. **(A)** A representative blot of pAkt and Akt in iPSC-derived non-CMS neurons (*n* = 3) and CMS neurons (*n* = 3) following hypoxia (1% O_2_) treatment for 0, 6, and 24 h. **(B)** Densitometry analysis of Akt. **(C)** A representative blot of Parkin in iPSC-derived non-CMS neurons (*n* = 3) and CMS neurons (*n* = 3) following hypoxia (1% O_2_) treatment for 0, 6, and 24 h. **(D)** Densitometry analysis of Parkin. **(E)** A representative blot of Mfn2 in iPSC-derived non-CMS neurons (*n* = 3) and CMS neurons (*n* = 3) following hypoxia (1% O_2_) treatment for 0, 6, and 24 h. Red line indicates the ubiquitylated Mfn2 (uMfn2). **(F)** Densitometry analysis of steady state of Mfn2 and **(G)** densitometry analysis of uMfn2. * indicates *p* < 0.05 and ** indicates *p* < 0.01 as compared to non-CMS neurons at a given time point. ^##^ indicates *p* < 0.01 as compared to themselves under normoxia.

Since (a) PERK is a direct Akt target and was hyperactivated in CMS neurons after hypoxia treatment (Blaustein et al., 2013), and (b) Akt has been shown to be associated with chronic mountain sickness (Buroker et al., 2017), we determined the level of Akt activation (pAkt S473/Akt) in both non-CMS and CMS neurons. As shown in Figures 2A,B, there was no difference between non-CMS neurons and CMS neurons in Akt activation (*p* > 0.05) at baseline. Hypoxia treatment induced Akt activation in non-CMS neurons significantly (Figures 2A,B, ##*p* < 0.01) but did not in CMS neurons. Interestingly, the difference of Akt activation between non-CMS and CMS was significant at 24 h (Figures 2A,B and ^∗∗^*p* < 0.01).

### Differences in Parkin Expression Between CMS and Non-CMS Neurons Under Hypoxia

Inhibition of Akt has been shown to affect Pink accumulation, and ubiquitination and Parkin recruitment in SHSY5Y cells and iPSC-derived neurons (Mccoy et al., 2014; Soutar et al., 2018). Both PINK1 and Parkin are important for mitochondrial quality control and mitophagy. Since the level of Akt activation in CMS neurons is significantly lower than that in non-CMS neurons under hypoxia, we then determined whether a lack of Akt activation in CMS affects PINK1 and Parkin expression. We first measured the expression of PINK1 and PINK1 substrate pS65-ubiquitin. Neither PINK1 nor PINK1 substrate pS65-ubiquitin expression were detected in non-CMS or CMS neurons (Supplementary Figure 1), suggesting the lack of PINK1-mediated ubiquitination in these neurons. Furthermore, we observed Parkin expression in both non-CMS and CMS neurons. As shown in Figures 2C,D, there was no difference in Parkin expression between non-CMS neurons and CMS neurons at baseline. Hypoxia alone did not change Parkin expression level in either non-CMS or CMS neurons (Figures 2C,D, *p* > 0.05). Of note, however, there was a difference in Parkin expression between non-CMS neurons and CMS neurons and this was significant at 24 h (Figures 2C,D, ^∗^*p* < 0.05), indicating that the changes of Parkin expression were not meditated through PINK1 signaling pathway.

### Hypoxia Decreases Mfn2 Expression in Non-CMS Neurons

Mfn2 is a tether protein for ER-mitochondria contact and can be regulated by both Akt and Parkin. Overexpression of active Akt was shown to down-regulate Mfn2 (Dasgupta et al., 2015) and decreased Parkin expression was shown to increase steady state Mfn2 expression (Gautier et al., 2016; Lee et al., 2019). We compared the expression of Mfn2 and found that the baseline level of Mfn2 was similar between non-CMS and CMS neurons; however, hypoxia significantly decreased Mfn2 steady state expression in non-CMS (Figures 2E,F, ##*p* < 0.01) but not in CMS neurons. Furthermore, the difference of Mfn2 expression between non-CMS neurons and CMS neurons was also significant at 24 h (Figures 2E,F, ^∗^*p* < 0.05). To determine whether a decreased Mfn2 steady state expression in non-CMS under hypoxia was due to an increased ubiquitination-mediated degradation, we compared the ratio of ubiquitylated Mfn2/non-ubiquitylated Mfn2 as described previously (Steffen et al., 2017; Mclelland et al., 2018). We found that non-CMS neurons have a significantly increased Mfn2 ubiquitination at 24 h (Figure 2G, ^∗^*p* < 0.05), suggesting the presence of a PINK1-independent ubiquitination.

### Enhanced Akt Activation and Parkin Overexpression Protect CMS Neurons From Hypoxia-Induced Cell Death

**FIGURE 3:**
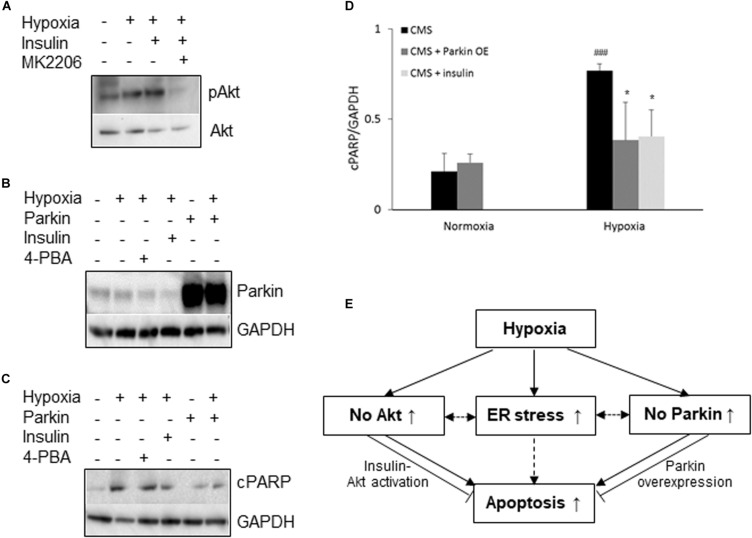
Neuroprotective role of Akt and Parkin in CMS. **(A)** A representative blot of pAkt in iPSC-derived CMS neurons (*n* = 3) following hypoxia (1% O_2_), hypoxia + 100 nM insulin and hypoxia + 100 nM insulin + MK2206 treatment for 48 h. **(B)** A representative blot of Parkin in iPSC-derived CMS neurons (*n* = 3) after a lentivirus transduction following hypoxia treatment or hypoxia with insulin or 4-PBA. **(C)** A representative blot of cPARP in iPSC-derived CMS neurons (*n* = 3) and Parkin-overexpressed CMS neuron (*n* = 3) following 1 mM 4-PBA, 100 nM insulin and hypoxia (1% O_2_) treatment for 48 h. **(D)** densitometry analysis of cPARP (4-PBA data was summarized in Figure 1H). * indicates *p* < 0.05 as compared to CMS neurons under hypoxia, ^###^*p* < 0.001 as compared to themselves under normoxia. **(E)** Schematic overview of current findings. Hypoxic treatment results in an increased ER stress response and lack of Akt activation and Parkin expression in CMS neurons. Failure to activate Akt and expression of Parkin render CMS neurons more susceptible to hypoxia-induced cell death, which can be alleviated by insulin-induced Akt activation and Parkin overexpression. Solid line indicates findings obtained from the current study, and dashed lines indicate findings from literature.

Since both Akt activation and Parkin overexpression are protective mechanisms, we then examined whether lack of Akt activation or Parkin overexpression in CMS neurons leads to an increased susceptibility to hypoxia injury and cell death, we treated CMS neurons with (1) Akt agonist (insulin) and (2) an overexpression of Parkin with hypoxia for 48 h. In these experiments, the expression level of cPARP was determined. Consistent with previous studies (Kim et al., 2011a; Zhao et al., 2017b), Akt activation was induced by insulin (∼2.1-fold increase, *p* < 0.05) and the Akt inhibitor MK2206 inhibits insulin-induced Akt activation (Figure 3A). Both increased Akt activation by insulin and overexpression of Parkin by lentivirus transduction (∼11-fold increase, *p* < 0.01, Figure 3B) significantly attenuated hypoxia-induced cell death in CMS neurons as compared to hypoxia exposure alone (Figures 3C,D, ^∗^*p* < 0.05), confirming the protective role of Akt activation and Parkin overexpression in cell survival under hypoxia. The results are schematically summarized in Figure 3E.

## Discussion

In the current study, we report that hypoxia treatment induced an increased Akt activation and a decreased Mfn2 expression in non-CMS neurons. In contrast, hypoxia treatment results in an increased ER stress response in CMS neurons and lack of Akt activation and Parkin expression as compared to non-CMS neurons. Intriguingly, we found that by enhancing Akt activation and overexpressing Parkin in CMS neurons, hypoxia-induced CMS neuronal cell death was reduced, suggesting an important role of Akt signaling and Parkin overexpression in survival under hypoxia.

Stable and continuous oxygen supply to tissues is essential for the functional integrity of cells and survival of mammals, including humans. At high altitude, hypobaric hypoxia drives insufficient oxygen delivery to tissue and can result in hypoxic tissues. Hypoxia exposure results in many pathological consequences including cognitive impairment and contributes to a functional decline during aging (Yaffe et al., 2011; Babbar and Agarwal, 2012; Yeo, 2019). Interestingly, the majority of highlanders are able to adapt well and are considered as “healthy control.” However, approximately 15–18% of highlanders cannot adapt to hypoxia and develop CMS, characterized by severe hypoxemia, excessive erythrocytosis and many neurologic manifestations including migraine, mental fatigue, confusion, and memory loss. Hence, CMS could be looked at as having manifestations of aging of high altitude in early adulthood (Sime et al., 1975).

Although the cellular mechanisms of CMS neuropathology remain largely unexplored, by using iPSC-derived neurons, we have previously demonstrated that CMS neurons have altered mitochondrial dynamics, fragmented mitochondria, a decreased ATP level and an increased susceptibility to hypoxia-induced cell death (Zhao et al., 2018). Of particular recent interest is that genomic analysis based on the same Andeans population from our laboratory has demonstrated that many ER stress-related genes are associated with hypoxia adaptation (Stobdan et al., 2017). Since the ER and mitochondria form a direct physical contact *via* mitochondria associated membrane (MAM), mitochondrial damage can lead to the activation of the ER stress response, and *vice versa*, ER stress can result in mitochondrial damage (Bouman et al., 2011; Senft and Ronai, 2015). Clinically, CMS patients experience exaggerated systemic oxidative-inflammatory-nitrosative stress (Bailey et al., 2019) and this is known to promote ER stress (Nakamura and Lipton, 2007; Eletto et al., 2014). Therefore, it is not surprising to find an increased expression of the ER stress marker Grp78 in CMS neurons even under normoxia. Under physiological conditions, Grp78 normally binds to ER proteins that constitute sensors for activation of three homeostatic pathways: IRE1, activating transcription factor-6 (ATF6) and PERK. The accumulation of unfolded proteins in the ER lumen during cellular stress conditions competes for Grp78 binding, and the dissociation of Grp78 from the sensor proteins initiates the ER stress signaling pathways aiming at removing the unfolded/misfolded proteins and alleviating stress to restore normal cellular function (Alder et al., 2005). Increased Grp78 is consistent with an accumulation of unfolded/misfolded proteins in the ER, and increased expression of XBP1s indicates that the IRE1α-mediated ER stress pathway was activated in CMS neurons. Previous studies have shown that overexpression of Grp78 may protect neurons and astrocytes from ischemic injury through activating either UPR/macroautophagy or Akt pro-survival pathway (Ouyang et al., 2011; Lu et al., 2015; Casas, 2017), but the prolonged UPR may lead to cell dysfunction and eventually cell death (Fernandez et al., 2015). Therefore, it is possible that overexpression of Grp78 might be one of the adaptive mechanisms in CMS neurons to protect themselves from an accumulated unfolded protein-induced toxicity. However, we cannot exclude that a higher baseline of Grp78 could indicate a higher steady state of ER stress in CMS neurons.

Hypoxia is known to induce ER stress through the accumulation of unfolded or misfolded proteins, both IRE1α–XBP1 and PERK arm of the ER stress response are activates and toward repair or removal of the unfolded protein by either autophagy, ER associated degradation (ERAD) or apoptosis (Romero-Ramirez et al., 2004; Mujcic et al., 2013). In support, we found that the expression of Grp78, XBP1s, and phosphorylation of PERK trend to increase under hypoxia, but no difference of Grp78 and XBP1s was observed between non-CMS neurons and CMS neurons. The phosphorylation of PERK was more pronounced in CMS neurons compared to non-CMS neurons, suggesting that hypoxia in CMS neurons results in greater activation of the PERK pathway. It is well known that activation of the PERK pathway induces expression of CCAAT/-enhancer-binding protein homologus protein (CHOP) and other pro-apoptotic genes that can mediate cell death (Hiramatsu et al., 2015). However, interestingly enough, we were not able to detect CHOP expression in either CMS or non-CMS neurons under normoxia or hypoxia conditions. Although both tunicamycin and thapsigargin as ER inducers induced CHOP expression in CMS and non-CMS neurons (Supplementary Figure 2), these results indicate that, the increased hypoxia-induced cell death in CMS neurons was not mediated by CHOP. Furthermore, we found that treatment with 4-PBA, an ER stress alleviator, has a tendency to rescue hypoxia-induced cell death (Figures 1G,H) suggesting that ER stress alone may not play a major role in an increased cell death in CMS neurons.

Hypoxia induces an increase in Akt activation in non-CMS neurons but not in CMS neurons. Akt is a pro-survival signal and deactivation of Akt is a mediator of cell death (Luo et al., 2003). Akt activation is increased by short-term exposure to ER stress but is decreased by long-term exposure to ER stress (Hosoi et al., 2007). Therefore, we speculate that a higher baseline of ER stress (prolonged) prevents Akt activation in CMS neurons after hypoxia treatment and the lack of Akt activation in CMS results in an increased cell death. Our data further confirmed that enhancing Akt activation with insulin in CMS neurons can rescue hypoxia-induced cell death. PERK is a direct target of Akt and Akt phosphorylates PERK at threonine 799 and then inactivates PERK signaling (Hosoi et al., 2007; Mounir et al., 2011). Inhibition of Akt persistently activates the PERK pathway (Blaustein et al., 2013). Therefore, increased Akt activation in non-CMS neurons may prevent PERK activation and hence supports the idea that there was no ER stress in non-CMS neurons. Buroker and his colleagues found that Akt is one of the genes that are associated with chronic mountain sickness in Tibetans (Buroker et al., 2017). Our genome analysis from Andeans also identified SGK3 as one of top genes that are associated with CMS (Stobdan et al., 2017). SGK3 contains the same consensus substrate motif with Akt and shares common substrate targets, indicating that both Akt and SGK3 could substitute for each other or work synergistically (Castel and Scaltriti, 2017; Wang et al., 2019). Indeed, Wang et al. demonstrated that SGK3 silencing can induce ER stress and PERK overexpression in the aromatase inhibitor-resistant cancer cell line (Wang et al., 2017). Therefore, Akt and SGK3 might be other signaling proteins besides ER stress that can promote PERK activation in CMS neurons after hypoxia treatment. Interestingly, CMS bone marrow mononuclear cells were reported to have an increased Akt expression that promotes erythroblasts survival and decreases apoptosis in CMS patients with excessive erythrocytosis (Zhao et al., 2017a). The differences of Akt expression between neurons and bone marrow mononuclear cells in the two studies might be tissue-specific, since CMS patients have distinguishable tissue specific pathologies, namely in the central nervous system and in red blood cells (Azad et al., 2016; Zhao et al., 2018).

ER stress can induce Parkin expression and this expression protects cells from ER stress-induced cell death (Bouman et al., 2011). However, we did not observe an increased Parkin expression in CMS neurons as a possible result of increased ER stress under hypoxia, suggesting the potential loss of Parkin protection. In contrast, the level of Parkin expression in CMS neurons is significantly decreased as compared to that in the non-CMS neurons under hypoxia. Parkin loss of function increases the sensitivity of cells to ER stress-induced mitochondrial dysfunction and Parkin overexpression can protect cells from ER stress-induced mitochondrial damage through the maintenance of mitochondrial integrity (Bouman et al., 2011). Therefore, we believe that a decreased Parkin expression in CMS neurons under hypoxia render CMS neurons more sensitive to stress. Parkin overexpression increases retinal ganglion cell (RGC) survival in glaucomatous rats and partially restores the dysfunctional mitophagy (Dai et al., 2018). Parkin overexpression also attenuates dopaminergic neurodegeneration induced by 1-methyl-4-phenyl-1, 2, 3, 6-tetrahydropyridine (MPTP) through protecting the integrity of mitochondria (Bian et al., 2012). Indeed, our data confirmed that CMS neurons with Parkin overexpression attenuated the hypoxia-induced cell death, which might be due to the maintenance of mitochondrial integrity in CMS neurons under hypoxia. Parkin is also considered as a tumor suppressor. One of the tumor suppression mechanisms is mediated through regulating the hypoxia inducible factor (HIF) signaling pathway. Parkin interacts with HIF-1α and promotes HIF-1α degradation through ubiquitination. In many types of cancers, Parkin expression is frequently down regulated and therefore stabilizes HIF-1α and promotes tumor metastasis and cancer development (Liu et al., 2017, 2018). In the current study, overexpression of Parkin in CMS neurons may promote HIF-1α degradation and therefore lead to alleviation of hypoxia-induced injury as compared to those without overexpression of Parkin.

Mfn2 has been implicated in regulating mitochondrial dynamics, ER-mitochondrial tethering, mitophagy, mitochondrial metabolism and respiratory function, thus impacting cell fate and homeostasis (Bach et al., 2003; Eura et al., 2003; Schrepfer and Scorrano, 2016). Except for embryonic stem cell-induced neurons, degradation of Mfn is not required for mitophagy (Ordureau et al., 2020). In the current study, hypoxia induced a decreased expression of Mfn2 and increased Akt activation in non-CMS neurons. Akt activation has been shown to down-regulate Mfn2 (Dasgupta et al., 2015), therefore, a decreased Mfn2 expression in non-CMS neurons might be due to Akt activation. Hypoxia treatment has been shown to result in an increased Akt activation, a decreased Mfn2 expression and inhibition of mitochondrial apoptosis pathway in pulmonary artery smooth muscle cells (Fang et al., 2016). In another study, H_2_O_2_-induced oxidative stress led to concurrent increased Mfn-2 expression and apoptosis with profound inhibition of Akt activation in cultured neonatal rat cardiomyocytes (Shen et al., 2007). These observations suggested that Mfn2 and Akt are inversely correlated and activation of Akt and decreased Mfn2 may protect cells from oxidative stress-induced cell death, similar to what we observed in non-CMS neurons.

Mfn2 is a tether protein for ER and mitochondria and Parkin regulates the ER-mitochondria contact *via* Mfn2 (Basso et al., 2018). Although both Mfn2 and Parkin have been shown to alter the ER-mitochondria coupling, it is still under debate whether the decreased Mfn2 and Parkin lead to a decrease or increase ER-mitochondrial coupling (De Brito and Scorrano, 2008; Schneeberger et al., 2013; Filadi et al., 2015; Gautier et al., 2016; Naon et al., 2016; Basso et al., 2018). The discrepancy between studies might be due to context and condition-specific factors. However, previous studies have demonstrated that decreased Mfn2 results in a reduced ER-mitochondria contact in neurons (Schneeberger et al., 2013; Gautier et al., 2016; Lee et al., 2018). For instance, Schneeberger et al. (2013) showed that ablation of Mfn2 in pro-opiomelanocortin neurons resulted in loss of ER-mitochondria contacts, reduced oxygen consumption and energy expenditure. Gautier et al. (2016) demonstrated that (1) ER-mitochondria contact and ER- to- mitochondrial Ca^2+^ transfer are enhanced in iPSC-derived neurons derived from patient with Parkin mutation, (2) Mfn2 expression is also increased in Parkin mutant neurons and Mfn2 down-regulation or the exogenous expression of Parkin can restore Ca^2+^ transients. In the current study, we observed a decrease in Mfn2 in the non-CMS neurons and we speculate that decreased Mfn2 may result in a decreased ER-mitochondrial contact and reduce energy expenditure, which might be one of adaptive mechanisms for non-CMS neurons surviving under hypoxia (Horscroft et al., 2017). It is worth noting that the decreased Mfn2 expression may also lead to mitochondrial fragmentation or increase ER stress (Filadi et al., 2018), therefore the precise role of Mfn2 in non-CMS needs to be further investigated.

Altogether, we report that increased Akt activation acts as a protective or adaptive mechanism to prevent hypoxia-induced cell death in non-CMS neurons. In contrast, increased ER stress response may render CMS neurons more sensitive to stress; and the lack of protective mechanisms such as Akt activation and Parkin expression results in an increased cell death in CMS neurons under hypoxia. Our data provide evidence that loss of endogenous adaptive cytoprotection mechanisms such as impaired ER stress response and mitochondrial function contribute, at least in part, to the neuropathology in CMS patients.

## Data Availability Statement

Requests to access the datasets should be directed to HZ, huzhao@ucsd.edu.

## Ethics Statement

The studies involving human participants were reviewed and approved by under protocols approved by the University of California San Diego and the Universidad Peruana Cayetano Heredia. The patients/participants provided their written informed consent to participate in this study.

## Author Contributions

HZ and GH designed the studies. HZ carried out experiments and analyzed data. HZ, JL, GS, and GH wrote and edited the manuscript. All authors contributed to the article and approved the submitted version.

## Conflict of Interest

The authors declare that the research was conducted in the absence of any commercial or financial relationships that could be construed as a potential conflict of interest.

## Supplementary Material

Supplementary Figure 1 PINK1 and pS65-ubiquitin were not detected in non-CMS and CMS neurons. A representative blot of PINK1 and pS65-ubiquitin (redline) in iPSC-derived non-CMS neurons and CMS neurons following hypoxia (1% O_2_) treatment for 0, 6, and 24 h, CCCP-treated PC3 cells as positive control.

Supplementary Figure 2 CHOP was not detected in non-CMS and CMS neurons. A representative blot of CHOP in iPSC-derived non-CMS and CMS neurons following hypoxia (1% O_2_) treatment for 0, 24, 48, and 96 h, thapsigargin (TG)-treated non-CMS neurons and tunicamycin (TM)-treated CMS neurons as positive control for ER stress and CHOP expression.
