# Supplementary figures and images for: Neuroprotective Role of Akt in Hypoxia Adaptation in Andeans
Source: Front Neurosci. 2021 Jan 15;14:607711. doi: 10.3389/fnins.2020.607711 (PMC7843528; doi:10.3389/fnins.2020.607711)

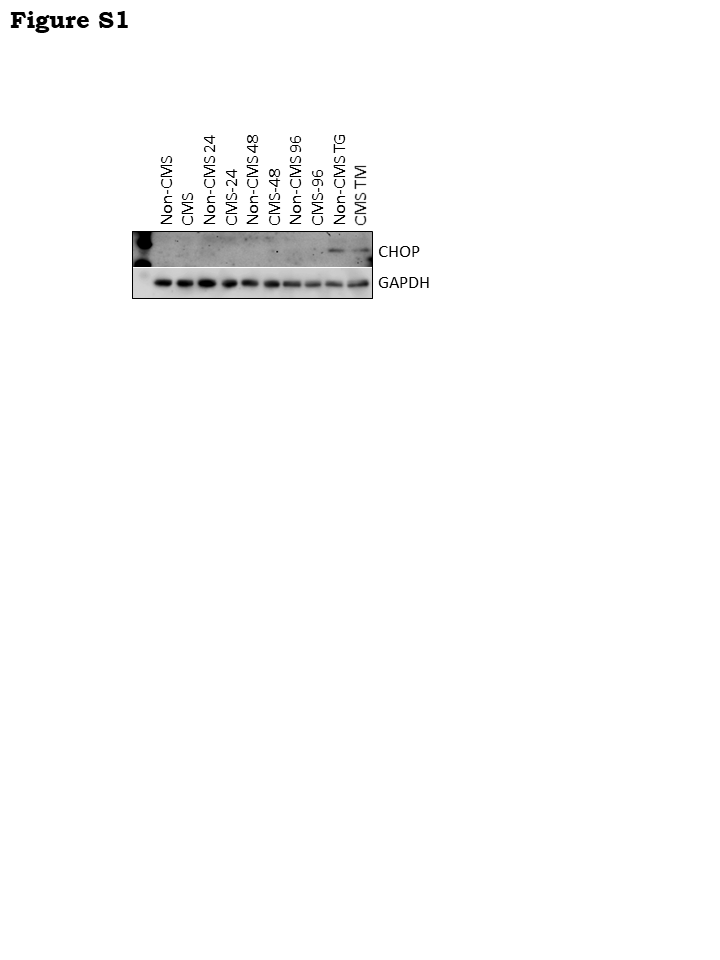

Supplement: Supplementary Figure 1 — PINK1 and pS65-ubiquitin were not detected in non-CMS and CMS neurons. A representative blot of PINK1 and pS65-ubiquitin (redline) in iPSC-derived non-CMS neurons and CMS neurons following hypoxia (1% O2) treatment for 0, 6, and 24 h, CCCP-treated PC3 cells as positive control. [file Image_1.tif]

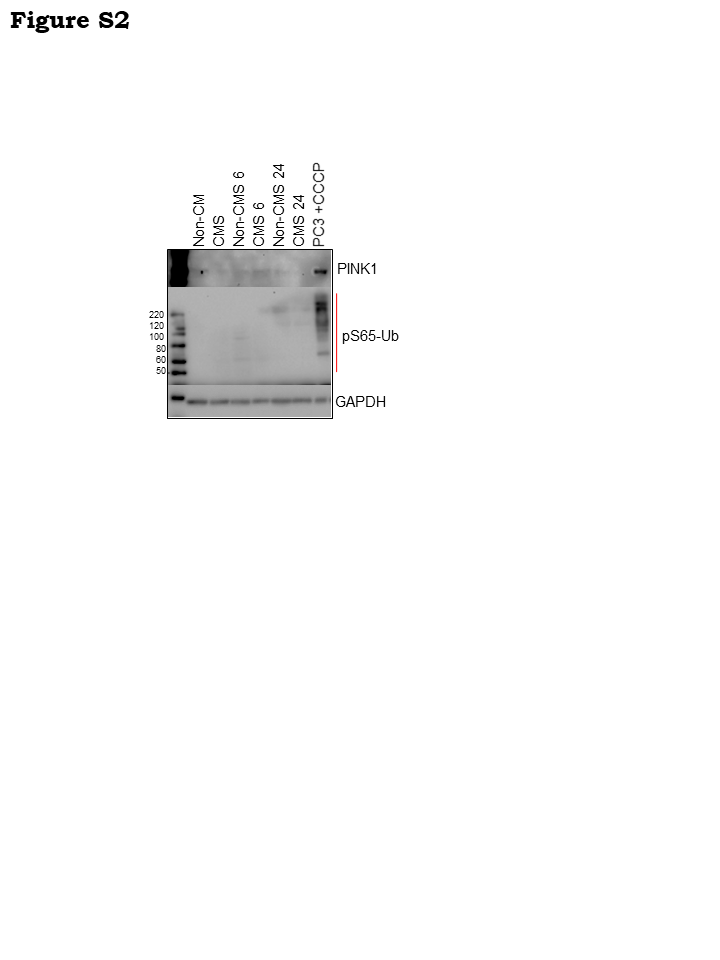

Supplement: Supplementary Figure 2 — CHOP was not detected in non-CMS and CMS neurons. A representative blot of CHOP in iPSC-derived non-CMS and CMS neurons following hypoxia (1% O2) treatment for 0, 24, 48, and 96 h, thapsigargin (TG)-treated non-CMS neurons and tunicamycin (TM)-treated CMS neurons as positive control for ER stress and CHOP expression. [file Image_2.tif]
